# The Abuse of Pituitrin, Intra-Partum

**Published:** 1915-11

**Authors:** F. J. Giuffrida

**Affiliations:** Atlanta, Ga.


### "THE ABUSE OF PITUITRIN, INTRA- PARTUM."

F. J. Giuffrida, M. D., Atlanta, Ga.

Just as we were about satisfied that ergot was dangerous during labor and had practically been set aside by obstetricians as a post-partum preparation only, a new and more valuable preparation was gotten up which acted quicker and more forcibly than argot,—namely; Putuitrin, the extract of the hypophyseal gland, which is used instead.

It is not my intention here to criticise this wonderful uterine stimulant, but I do want to show the abuse of it and discourage the idea of its taking the place of the judicious use of forceps. In my opinion, Pituitrin has no place in labor, but has no equal when indicated after the third stage, or just before opening the uterus in Caesarean section to insure firm contraction while closing same.

In this day, when the general practitioner has such limited time to spend with an obstetrical case, he is prone to become restless unless his patient gets through within a comparatively short time. Right here is where the trouble begins. He may have neglected to make himself familiar with the passages and the passenger. He may not have listened to the fetal heart. The danger of injury to the bladder may have passed out of his mind. Bandl's ring, the already fatigued fibres of the uterus and the thin lower segment are entirely overlooked in his haste to get through. It is very easy to see how much damage can be done if you just stop to think of these few but most important points. It is nothing less than meddlesome midwifery to employ pituitrin, or any other uterine stimulant just because we are tired hanging around a patient. This needless hurry, and the giving of intra-partum pituitrin, has made more wrecks of women than we have stopped to consider. Ruptured uterus, badly torn cervix and perineum, post-partum haemorrhage, stripping the bladder from its peritoneal attachment and asphyxia of the child;—any or all of these may be some of the calamities that may turn the tide of a normal labor into a most pathological condition, if you let haste get the better of your good judgment.

When the gynecologist gets this patient, it will reflect upon your reputation and refined feelings, if nothing worse. Consult authorities regarding the use of pituitrin intra-partum. Don't listen to what someone else did, who wanted to get a case over in the least possible time.

I don't think an obstetrical grip is complete without pituitrin, but please let us try not to abuse it.

1110 Candler Bldg.

